# A risk prediction model based on machine learning for early cognitive impairment in hypertension: Development and validation study

**DOI:** 10.3389/fpubh.2023.1143019

**Published:** 2023-03-09

**Authors:** Xia Zhong, Jie Yu, Feng Jiang, Haoyu Chen, Zhenyuan Wang, Jing Teng, Huachen Jiao

**Affiliations:** ^1^Department of First Clinical Medical College, Shandong University of Traditional Chinese Medicine, Jinan, Shandong, China; ^2^Shandong University of Traditional Chinese Medicine, Jinan, Shandong, China; ^3^Department of Cardiology, Affiliated Hospital of Shandong University of Traditional Chinese Medicine, Jinan, Shandong, China

**Keywords:** hypertension, cognitive impairment, machine learning, prediction model, risk factors

## Abstract

**Background:**

Clinical practice guidelines recommend early identification of cognitive impairment in individuals with hypertension with the help of risk prediction tools based on risk factors.

**Objective:**

The aim of this study was to develop a superior machine learning model based on easily collected variables to predict the risk of early cognitive impairment in hypertensive individuals, which could be used to optimize early cognitive impairment risk assessment strategies.

**Methods:**

For this cross-sectional study, 733 patients with hypertension (aged 30–85, 48.98% male) enrolled in multi-center hospitals in China were divided into a training group (70%) and a validation group (30%). After least absolute shrinkage and selection operator (LASSO) regression analysis with 5-fold cross-validation determined the modeling variables, three machine learning classifiers, logistic regression (LR), XGBoost (XGB), and gaussian naive bayes (GNB), were developed. The area under the ROC curve (AUC), accuracy, sensitivity, specificity, and F1 score were used to evaluate the model performance. Shape Additive explanation (SHAP) analysis was performed to rank feature importance. Further decision curve analysis (DCA) assessed the clinical performance of the established model and visualized it by nomogram.

**Results:**

Hip circumference, age, education levels, and physical activity were considered significant predictors of early cognitive impairment in hypertension. The AUC (0.88), F1 score (0.59), accuracy (0.81), sensitivity (0.84), and specificity (0.80) of the XGB model were superior to LR and GNB classifiers.

**Conclusion:**

The XGB model based on hip circumference, age, educational level, and physical activity has superior predictive performance and it shows promise in predicting the risk of cognitive impairment in hypertensive clinical settings.

## 1. Introduction

Hypertension has been recognized as a significant risk factor for cognitive impairment, which may increase the risk of vascular dementia and Alzheimer's events (1). According to recent evidence, hypertension was associated with a 1.86-fold and 1.62-fold increased risk of dementia and mild cognitive impairment in the Chinese population (2). Although the mechanism of these deleterious effects is poorly supported by conclusive evidence, preclinical investigations have provided potential mechanistic evidence for better insight. Chronic hypertension can continuously damage the structure and function of cerebral vessels, challenge the integrity of the blood-brain barrier through inflammatory pathologic pathways (3), and also promote the formation of atherosclerotic plaques and evolve into ischemic stroke (4), which is an important pathological basis for cognitive impairment (5). Although these possible mechanisms have given promising hints for the prevention and treatment of hypertensive cognitive impairment, this requires rigorous investigation to be confirmed in the future. Well-developed preventive procedures can significantly reduce the treatment burden of cognitive impairment in hypertensive populations. Maintaining cognitive health and preventing early cognitive impairment in hypertensive individuals is a critical public health priority. Therefore, it is necessary to investigate the risk factors of early cognitive impairment in the hypertensive population, establish an early risk prediction model, and explore its pathogenesis, to provide better decision-making for early cognitive impairment.

The traditional approach to the diagnosis of cognitive impairment in hypertension focuses on cognitive and neuropsychological assessment but is often criticized for its lag (6). More recently, although amyloid proteins, tau proteins, and several structural magnetic resonance imaging (MRI) indicators have been recognized as promising pathologic markers (7, 8), high costs and complex inspection procedures still limit their widespread use. Considering the multi-factorial characteristics of hypertensive cognitive impairment, it is necessary to combine multiple parameters to better reflect its pathological development. In recent years, relevant influencing factors of early cognitive impairment, including age, education, chronic disease, and modifiable life factors, as well as their independent effects and interactions, have been considered. Several observational studies have identified several potentially modifiable risk factors for cognitive decline, including hypertension, dyslipidemia and obesity, diabetes mellitus, alcohol consumption, smoking, physical inactivity, dietary habits such as sodium intake (9), and sensory function. Several previous studies have reported strong associations between plant-based diets (10), age-related central auditory processing disorder (CAPD) (11), and antihypertensive medications (12, 13) with cognitive decline.

In recent years, many researchers, especially Chinese, have studied cognitive impairment with hypertension, but most of them are limited to risk factors. The conclusion is controversial, and the number of prediction models is limited. A recent community survey from China showed that hypertension grade, smoking, sleep disorder, and duration of hypertension were risk factors, while education, exercise, reading, social support, and medication adherence were protective factors; AUC, sensitivity, and specificity of the model developed based on influencing factors were 0.765, 0.630, and 0.877 (14). Another study based on hypertensive patients from China showed that duration of hypertension, SBP, homocysteine (Hcy), and SUA were risk factors for developing cognitive dysfunction, and duration of education was a protective factor for developing cognitive dysfunction (15). In addition, Zhang et al. (16) conducted a study based on the hypertensive population in plateau areas of China indicated that plateau environment, age, abdominal circumference, and SUA are independent risk factors affecting hypertensive cognitive impairment. Ma et al. (17) reported that low education attainment and elevated BMI, WHR, and homeostasis assessment model for insulin resistance index (HOMA-IR) are independent risk factors for cognitive impairment in elderly patients with hypertension. Qu et al. (18) performed a cohort study to reveal that intestinal microbiota dysbiosis may be an important predictor of cognitive impairment with hypertension.

With the development of artificial intelligence, machine learning techniques have been used in cardiovascular event risk prediction models to improve accuracy and other performance (19–21), providing a new paradigm for cardiovascular monitoring. However, the risk prediction model for early cognitive impairment in hypertensive populations based on machine learning has never been reported. Accordingly, we developed a predictive machine learning model that considers the independent effects and interactions of influencing factors to assess the risk of early cognitive impairment in the Chinese hypertensive population, which would conduct early risk screening strategies and interventions for hypertensive cognitive impairment. Here, we hypothesized that machine learning could be used to diagnose early cognitive impairment based on the clinical characteristics of individuals with hypertension.

## 2. Materials and methods

### 2.1. Study design and participants

We conducted a multicenter observational study of hospitalized hypertensive patients, which considered geographic region, urbanization, gender, and age distribution. For this cross-sectional study, we randomly selected 5 prefecture-level cities in Shandong Province by stratified cluster sampling, including Jinan, Yantai, Weifang, Dongying, and Jining, and then randomly selected 8 hospitals in the selected prefecture-level cities. All patients with hypertension in hospitals were selected for this study, and 787 individuals were recruited from May 2022 to December 2022. This study was approved by the Institutional Review Committee (IRB) of Affiliated Hospital of the Shandong University of Chinese Medicine and obtained the informed consent of all study participants. All participants signed informed consent. Individuals over 30 years of age with essential hypertension were included in this study. Meanwhile, we excluded patients >85 years of age with a history of stroke, Parkinson's disease, brain trauma, brain tumor, epilepsy, vision or hearing impairment, dementia, mental or psychiatric illness, severe impairment of heart, liver, or kidney function, combined with severe infection, tumor, hyperthyroidism, heart failure, arrhythmia, or cardiac surgery. In addition, we excluded 16 patients with missing data, 8 patients with abnormal data, and 6 patients with MMSE scores <18 points, leaving 733 samples for analysis.

### 2.2. Sample size calculation

According to previous reports (22, 23), the incidence of cognitive impairment in the Chinese hospitalized hypertensive population π_0_ = 0.25, α = 0.05, β = 0.10, allowable error (δ) = 0.10, Z_β_ = 1.282 beta, Z_α_ = 1.960, *n* = ((Z_β_+*Z*_α_)/δ)^2^ × π_0_ × (1- π_0_), two-tailed test. According to the formula, the calculated sample size was at least 197 patients. Considering the loss of follow-up rate, a total of 733 patients were finally included in this study.

### 2.3. Predictors

Two sets of predictors (easy to collect variables, including socio-demographics, lifestyle factors, family history, laboratory test parameters, imaging parameters, and drug information) were considered for machine learning model development. Socio-demographic, lifestyle factors, family history, and medication information for all patients were obtained through questionnaires. Data collected included sex, age, marital status, educational level, smoking status, drinking status, type of work, estimated duration of hypertension, average salt intake per month, and medication information. Sleep parameters including night sleep onset time, night sleep duration, night sleep latency, and PSQI score were obtained by the PSQI questionnaire. PSQI is a reliable self-report tool used to assess patients' sleep quality over the past month (24), and its results involve scores on seven components, including sleep quality, sleep latency, sleep duration, sleep efficiency, sleep disorders, sleep medications, and daytime sleep disorders (25). Physical activity was obtained through the international physical activity questionnaire (IPAQ). The IPAQ (long form) consists of 27 questions about subjects' activities during the last 7 days as follows (26): (1) professional sports activities; (2) transportation sports activities; (3) housework, house maintenance, and family care; (4) recreation, sports, and leisure sports activities; (5) sitting time. Blood pressure measurements for all participants were taken during a single visit. Systolic blood pressure (SBP) and diastolic blood pressure (DBP) were measured at 2-min intervals, and the average of the three measurements was calculated consecutively (27). Anthropometric variables, including height (in centimeters), weight (in kilograms), waist circumference (in centimeters), and hip circumference (in centimeters) measurements of all participants were measured using standardized techniques and equipment by two trained interviewers; body mass index (BMI) was calculated by dividing weight in kilograms by height in meters squared (kg/m2) (28). Fasting blood glucose (FBG), triglyceride (TG), total cholesterol (TC), low-density lipoprotein-cholesterol (LDL-C), and serum creatinine (SCr) were collected from laboratory tests by professional physicians. Right atrial diameter (RAD), left atrial diameter (LAD), right ventricular diameter (RVD), and left ventricular diameter (LVD) were measured by experienced cardiac color ultrasound physicians.

### 2.4. Diagnostic criteria

Two experienced cardiologists assessed hypertension diagnosis using the following criteria (29): SBP ≥ 140 mmHg, DBP ≥90 mmHg, and/or the use of antihypertensive drugs. Two other trained investigators used MMSE to assess the diagnosis of early cognitive impairment within 5–10 min. The Chinese version of MMSE has been used for the early cognitive assessment of all individuals, which has been shown to be effective and reliable in the Chinese population (30). MMSE covers simple task areas: time and place, repetitive words, arithmetic, language, and motor skills, with a total of 30 scores (31). MMSE scores above 18 and below 27 were defined as early cognitive impairment, and MMSE scores above 27 were considered normal cognitive function (32, 33).

### 2.5. Outcomes

A total of 122 (16.64%) participants had a diagnosis of cognitive impairment in 733 hypertensive individuals. 16 core variables were selected from 35 conventional variables for LASSO regression analysis, including 4 sociodemographic factors, 6 lifestyle factors, 1 laboratory test parameter, 3 imaging parameters, and 2 medication factors. Finally, four predictive variables, including age, hip circumference, education levels, and physical activity, were selected for the development of machine learning models.

### 2.6. Statistical methods

All statistical analyses in the current study were performed using R version 3.6.3 and Python version 3.7. Continuous variables were expressed by mean [standard deviation (SD)] or median [25th, 75th], and categorical variables were expressed by number (percentage%). To ensure the simplicity of the model, we performed *T*-tests, Mannwhitney-U tests, and Chi-square tests to screen for variables with statistical differences between the non-MCI group and the MCI group, and further least absolute shrinkage and selection operator (LASSO) analysis with 5-fold cross-validation was performed for dimension reduction to filter the most suitable predictors to build the machine learning model. The selected individuals in the current study were randomly divided into a training set and a validation set (7:3), and analyzed by three classifiers (LR, XGB, and GNB). By comparing their AUC, accuracy, sensitivity, specificity and F1 score, the prediction model with the most perfect prediction performance was selected. The ROC curve was developed to obtain the AUC of the predictive model, and its predictive power was further evaluated by calibrating the curve. Shape Additive Explanation (SHAP) analysis was applied to investigate the model's feature importance, while the DCA curve was developed to evaluate the model's clinical applicability. If a *p*-value in 2-sided tests is <0.05, it is considered statistically significant.

### 2.7. Machine learning models

**Figure 1:**
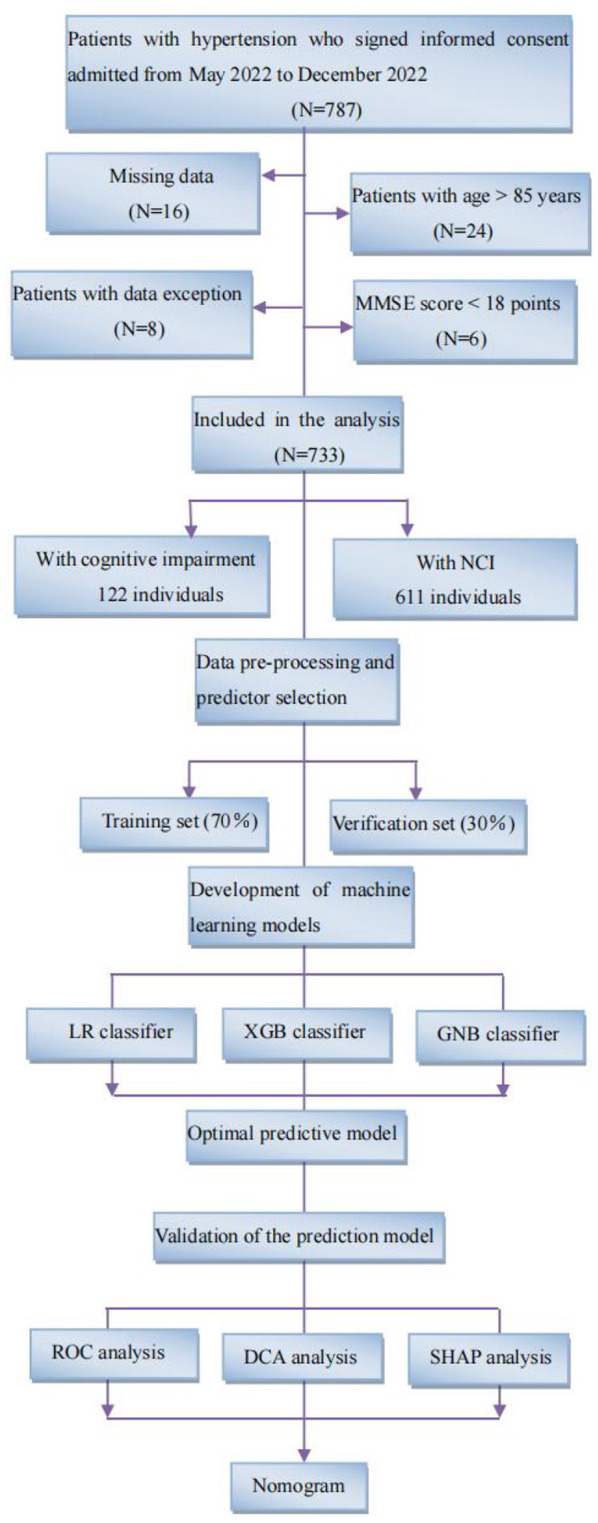
Study flow diagram. Flowchart illustrating patient selection and machine learning model development pipeline. Following standard inclusion and exclusion procedures, a total of 733 individuals were selected, including 122 patients with cognitive impairment and 611 NCI. We developed machine learning models using three classifiers, LR, XGB, and GNB, and synthesized them into an integrated model. All individuals were randomly assigned to one of two groups: 70% training and 30% verification. Five-fold cross-validation (CV) was used to train and verify the model for 10 repetitions. MMSE, mini-mental state examination; NCI, no cognitive impairment; LR, logistic regression; XGB, XGBoost; GNB, gaussian naive bayes; ROC, receiver operating characteristic; SHAP, shape additive explanation.

Figure 1 illustrates the machine learning model development pipeline. The current models were developed by three classifiers, including logistic regression (LR), XGBoost (XGB), and gaussian naive bayes (GNB). Finally, we select the model with the best predictive performance according to the predictive performance of the three classifiers. Based on selected variables, we randomly divided the individuals into two groups: 70% training for model development and hyperparameter tuning and 30% verification for model evaluation. The model was trained and verified for 10 repetitions using five-fold cross validation (CV). AUC, accuracy, specificity, sensitivity, and F1 scores were used to evaluate the performance of the machine learning models. The classification confusion matrix definition is that individuals with cognitive decline are considered true positive (TP) and true negative (TN) if they are accurately predicted by the machine learning model; In contrast, it is considered false positive (FP) or false negative (FN) (6). AUC, the area under the ROC curve, the larger the value, the better the classification effect. Accuracy is defined as the proportion of correctly classified samples to total samples for a given data, which can be calculated by the following formula: Accuracy = (TP+TN)/(TP+TN+FP+FN). Sensitivity refers to the percentage of samples that are positively determined to be positive, which can be calculated by the following formula: Sensitivity = TP/(TP+FN). Specificity refers to the percentage of samples that are actually negative that are determined to be negative, which can be calculated by the following formula: Specificity = TN/(TN+FP). Precision and recall are two commonly used evaluation indexes for the binary classification problems, in which precision refers to the proportion of real class in the predicted positive class sample, and recall refers to the proportion of predicted positive class in all the predicted positive class samples. F1-score is the evaluation standard to measure the comprehensive performance of classifiers, which can be calculated by the following formula: F1 score = 2 x precision x recall/(precision + recall).

## 3. Results

### 3.1. Comparison of demographic and clinical characteristics between early cognitive impairment and NCI

Table 1 shows the demographic and clinical characteristics of all participants. The mean age of the individuals was 66.37 (10.88) years and 48.98% of the individuals were male. A total of 122 (16.64%) participants had a diagnosis of early cognitive impairment. Compared with NCI, patients with early cognitive impairment were found to be older [mean age 74.60 (7.28 years)], lower educational attainment, longer duration of hypertension, waist circumference, hip circumference, poorer sleep quality, less physical activity, higher levels of Scr, larger RAD, LAD, LVD, less likely to use ACEI/ARBs and beta-blockers (all *p* < 0.05). However, preliminary analysis showed no statistical difference between the two groups in gender, smoking, alcohol consumption, marital status, average salt intake per month, blood pressure level, BMI, night sleep duration, FBG, lipid profiles, RVD, family history, CCBs, and diuretic use (all *p* > 0.05).

**Table 1:** Comparison of demographic and clinical characteristics between cognitive impairment and NCI.

**Variables**	**Overall (*N =* 733)**	**NCI (*N =* 611)**	**Early cognitive impairment (*N =* 122)**	***P*-value**
Age, years, mean (SD)	66.37 (10.88)	64.72 (10.74)	74.60 (7.28)	<0.001^*^
Sex (Male), *n* (%)	359 (48.98)	300 (49.10)	59 (48.36)	0.881
Current smoker, *n* (%)	225 (30.70)	194 (31.75)	31 (25.41)	0.166
Current drinker, *n* (%)	189 (25.78)	163 (26.68)	26 (21.31)	0.216
**Marital status**, ***n*** **(%)**				
Married	713 (97.27)	594 (97.22)	119 (97.54)	0.841
Unmarried, divorced or widowed	20 (2.73)	17 (2.78)	3 (2.46)	
**Educational levels**, ***n*** **(%)**				<0.001^*^
Primary school or below	285 (38.88)	200 (32.73)	85 (69.67)	
Junior high school or senior high school	402 (54.84)	365 (59.74)	37 (30.33)	
University or above	46 (6.28)	46 (7.53)	0 (0.00)	
**Type of work**, ***n*** **(%)**				<0.001^*^
Manual labor	392 (53.48)	297 (48.61)	95 (77.87)	
Mental labor	101 (13.78)	98 (16.04)	3 (2.46)	
Both manual and brain labor	240 (32.74)	216 (35.35)	24 (19.67)	
Estimated duration of hypertension, months, median [IQR]	115 [59.00,160.00]	107 [57.00,156.00]	131 [67.00,196.00]	0.018^*^
Average salt intake per month, g, median [IQR]	300 [180.00, 600.00]	300 [180.00, 600.00]	300 [240.00, 480.00]	0.152
SBP, mmHg, mean (SD)	142.76 (14.73)	142.24 (14.08)	145.39 (17.49)	0.063
DBP, mmHg, mean (SD)	83.69 (12.94)	83.88 (13.42)	82.77 (10.19)	0.301
Waist circumference, cm, mean (SD)	84.21 (15.32)	83.35 (15.74)	88.51 (12.18)	<0.001^*^
Hip circumference, cm, mean (SD)	97.67 (12.34)	96.78 (12.35)	102.14 (11.35)	<0.001^*^
BMI, kg/m^2^, mean (SD)	24.99 (3.31)	24.90 (3.32)	25.47 (3.23)	0.082
**Sleep parameters**				
Night sleep initiation time, hour, mean (SD)	21.68 (0.91)	21.75 (0.94)	21.38 (0.73)	<0.001^*^
Night sleep duration, hours, mean (SD)	7.11 (0.89)	7.14 (0.86)	6.98 (1.04)	0.113
Night sleep latency, minutes, median [IQR]	20 [10.00, 30.00]	20 [10.00, 30.00]	30 [20.00, 30.00]	<0.001^*^
PSQI score, points, mean (SD)	6.11 (3.87)	5.79 (3.97)	7.75 (2.83)	<0.001^*^
**Physical activity**, ***n*** **(%)**				<0.001^*^
Light	143 (19.51)	101 (16.53)	42 (34.43)	
Moderate	399 (54.43)	330 (54.01)	69 (56.56)	
Vigorous	191 (26.06)	180 (29.46)	11 (9.02)	
**Laboratory testing parameters**				
FBG, mmol/L, mean (SD)	6.54 (2.10)	6.50 (2.09)	6.71 (2.16)	0.314
TG, mmol/L, mean (SD)	1.68 (1.28)	1.70 (1.34)	1.54 (0.85)	0.090
TC, mmol/L, mean (SD)	4.64 (1.27)	4.68 (1.28)	4.44 (1.22)	0.057
LDL-C, mmol/L, mean (SD)	2.73 (1.02)	2.76 (1.03)	2.57 (0.95)	0.060
SCr, μmoI/L, median [IQR]	66 [55.00, 78.30]	65 [54.70, 77.00]	71 [60.30, 88.00]	<0.001^*^
**Imaging parameters**				
RAD, mm, mean (SD)	33.13 (5.75)	32.84 (5.57)	34.57 (6.38)	0.006^*^
LAD, mm, mean (SD)	36.65 (5.92)	36.24 (5.72)	38.73 (6.50)	<0.001^*^
RVD, mm, mean (SD)	21.97 (3.18)	21.85 (3.00)	22.57 (3.91)	0.056
LVD, mm, mean (SD)	47.51 (6.51)	47.15 (6.58)	49.29 (5.85)	0.001^*^
**Family history**, ***n*** **(%)**				
Family history of hypertension	446 (60.85)	380 (62.19)	66 (54.10)	0.094
Family history of CHD	203 (27.69)	173 (28.31)	30 (24.59)	0.401
Family history of hyperlipemia	169 (23.06)	145 (23.73)	24 (19.67)	0.331
**Medication information**, ***n*** **(%)**				
CCBs use	443 (60.44)	366 (59.90)	77 (63.12)	0.508
ACEI/ARBs use	472 (64.39)	407 (66.61)	65 (53.28)	0.005^*^
Beta-blockers use	139 (18.96)	124 (20.30)	15 (12.30)	0.040^*^
Diuretics use	128 (17.46)	106 (17.35)	22 (18.03)	0.856

Data are presented as mean (SD), median [IQR], or n (%).

NCI, no cognitive impairment; SBP, systolic blood pressure; DBP, diastolic blood pressure; BMI, body mass index; PSQI, Pittsburgh Sleep Quality Index; FBG, fasting blood glucose; TG, triglyceride; TC, total cholesterol; LDL-C, low-density lipoprotein cholesterol; SCr, serum creatinine; RAD, right atrial diameter; LAD, left atrial diameter; RVD, right ventricular diameter; LVD, left ventricular diameter; CHD, coronary heart disease; CCB, calcium channel blocker; ACEI, angiotensin-converting enzyme inhibitors; ARB, angiotensin receptor blocker.

* Statistically significant (P < 0.05).

### 3.2. Comparison of demographic and clinical characteristics between training and verification sets

Table 2 shows the demographic and clinical characteristics between the training set and the verification set. A total of 513 people were included in this study as the training set and 220 as the test set, with a ratio of 7:3. Current results indicate no statistical difference in most predictive variables between the training set and verification set (all *P* > 0.05).

**Table 2:** Comparison of demographic and clinical characteristics between training and verification sets.

**Variables**	**Training set (*N =* 513)**	**Verification set (*N =* 220)**	***P*-value**
Age, years, mean (SD)	66.07 (10.70)	67.06 (11.29)	0.259
Sex (Male), *n* (%)	236 (46.00)	123 (55.91)	0.014
Current smoker, *n* (%)	156 (30.41)	69 (31.36)	0.797
Current drinker, *n* (%)	134 (26.12)	55 (25.00)	0.751
**Marital status**, ***n*** **(%)**			0.620
Married	498 (97.08)	215 (97.73)	
Unmarried, divorced or widowed	15 (2.92)	5 (2.27)	
**Educational levels**, ***n*** **(%)**			0.274
Primary school or below	192 (37.43)	93 (42.27)	
Junior high school or senior high school	285 (55.56)	117 (53.18)	
University or above	36 (7.02)	10 (4.55)	
**Type of work**, ***n*** **(%)**			0.029
Manual labor	260 (50.68)	132 (60.00)	
Mental labor	80 (15.60)	21 (9.55)	
Both manual and brain labor	173 (33.72)	67 (30.46)	
Estimated duration of hypertension, months, median [IQR]	113 [57.00, 179.00]	117 [62.00, 152.00]	0.784
Average salt intake per month, g, median [IQR]	300 [180.00, 600.00]	300 [180.00, 600.00]	0.570
SBP, mmHg, mean (SD)	142.78 (14.98)	142.72 (14.17)	0.960
DBP, mmHg, mean (SD)	83.38 (12.82)	84.41 (13.22)	0.324
Waist circumference, cm, mean (SD)	83.51 (15.44)	85.85 (14.93)	0.058
Hip circumference, cm, mean (SD)	97.67 (12.37)	97.68 (12.30)	0.992
BMI, kg/m^2^, mean (SD)	24.84 (3.30)	25.35 (3.32)	0.056
**Sleep parameters**			
Night sleep initiation time, hour, mean (SD)	21.70 (0.91)	21.65 (0.91)	0.496
Night sleep duration, hours, mean (SD)	7.11 (0.88)	7.11 (0.92)	1.000
Night sleep latency, minutes, median [IQR]	20 [10.00, 30.00]	20 [10.00, 30.00]	0.510
PSQI score, points, mean (SD)	6.10 (3.85)	6.14 (3.93)	0.898
**Physical activity**, ***n*** **(%)**			0.588
Light	104 (20.27)	39 (17.73)	
Moderate	280 (54.58)	119 (54.09)	
Vigorous	129 (25.15)	62 (28.18)	
**Laboratory testing parameters**			
FBG, mmol/L, mean (SD)	6.49 (2.01)	6.65 (2.30)	0.371
TG, mmol/L, mean (SD)	1.67 (1.21)	1.70 (1.41)	0.783
TC, mmol/L, mean (SD)	4.66 (1.28)	4.59 (1.24)	0.494
LDL-C, mmol/L, mean (SD)	2.75 (1.02)	2.70 (1.02)	0.543
SCr, μmoI/L, median [IQR]	65 [54.70, 78.00]	67.30 [56.00, 80.56]	0.206
**Imaging parameters**			
RAD, mm, mean (SD)	33.13 (5.80)	33.11 (5.64)	0.966
LAD, mm, mean (SD)	36.90 (6.20)	36.08 (5.19)	0.066
RVD, mm, mean (SD)	22.00 (3.28)	22.89 (2.94)	0.001^*^
LVD, mm, mean (SD)	47.50 (6.37)	47.54 (6.85)	0.939
**Family history**, ***n*** **(%)**			
Family history of hypertension	309 (60.23)	137 (62.27)	0.604
Family history of CHD	141 (27.49)	62 (28.18)	0.847
Family history of hyperlipemia	120 (23.39)	49 (22.27)	0.742
**Medication information**, ***n*** **(%)**			
CCBs use	315 (61.40)	128 (58.18)	0.414
ACEI/ARBs use	333 (64.91)	139 (63.18)	0.654
Beta-blockers use	100 (19.49)	39 (17.73)	0.576
Diuretics use	84 (16.37)	44 (20.00)	0.236

Data are presented as mean (SD), median [IQR], or n (%).

NCI, no cognitive impairment; SBP, systolic blood pressure; DBP, diastolic blood pressure; BMI, body mass index; PSQI, Pittsburgh Sleep Quality Index; FBG, fasting blood glucose; TG, triglyceride; TC, total cholesterol; LDL-C, low-density lipoprotein cholesterol; SCr, serum creatinine; RAD, right atrial diameter; LAD, left atrial diameter; RVD, right ventricular diameter; LVD, left ventricular diameter; CHD, coronary heart disease; CCB, calcium channel blocker; ACEI, angiotensin-converting enzyme inhibitors; ARB, angiotensin receptor blocker.

* Statistically significant (P < 0.05).

### 3.3. Screening of modeling variables based on LASSO regression analysis

Here, we used LASSO regression to screen and reduce the dimension of the 16 variables with statistical differences in Table 1, including age, educational level, type of work, duration of hypertension, waist circumference, hip circumference, night sleep initiation time, night sleep latency, PSQI score, physical activity, Scr, RAD, LAD, LVD, ACEI/ARB use, and beta-blockers. As log (λ) increases, the average standard error increases, and the normalization coefficients of the 16 candidate variables are compressed to varying degrees until all of them become zero (34). Current results show that when the lambda of the minimum standard error was 0.05, the continuous variables of the gaussian model were selected as hip circumference and age; when the lambda of the minimum standard error was 0.038, the classification variables of the binomial model were physical activity and educational levels. Finally, we determined four predictive variables for machine learning modeling, including hip circumference, age, education level, and physical activity.

### 3.4. Development of a predictive machine learning model

Table 3 shows the performance of the prediction model. Current analysis shows that the best performance model was the XGB model, with an AUC of 0.88, accuracy of 0.81, sensitivity of 0.84, specificity of 0.80, and F1 score of 0.59. The second model was the LR model, with an AUC of 0.83, accuracy of 0.740, sensitivity of 0.78, specificity of 0.73, and F1 score of 0.50. Compared to the XGB model and LR model, the GNB model had poor performance, with an AUC of 0.816, accuracy of 0.74, sensitivity of 0.75, specificity of 0.74, and F1 score of 0.50.

**Table 3:** Performance of the developed models based on four classifiers.

**Classifiers/performance**	**AUC (95%CI)**	**Accuracy**	**Sensitivity**	**Specificity**	**F1 score**
LR model	0.83 (0.79–0.87)	0.74	0.78	0.73	0.50
XGB model	0.88 (0.85–0.91)	0.81	0.84	0.80	0.59
GNB model	0.82 (0.78–0.86)	0.74	0.75	0.74	0.50

AUC, area under the curve; LR, logistic regression; XGB, XGBoost; GNB, gaussian naive bayes.

### 3.5. Evaluation of machine learning prediction model based on XGB

**Figure 2:**
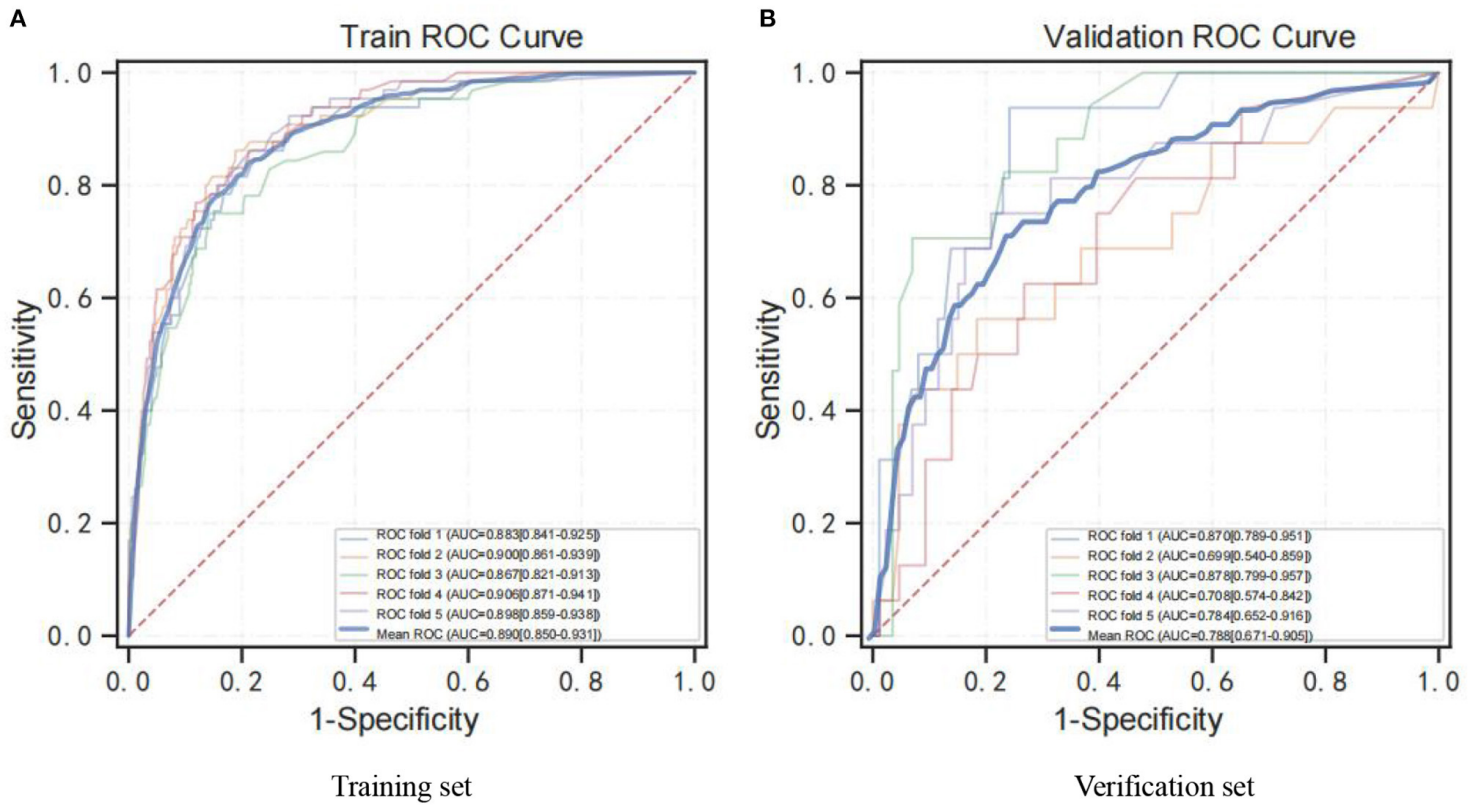
ROC curve for the XGB model. **(A)** ROC analysis results of the XGB model based on training set data by 5-fold cross-validation. **(B)** ROC analysis results of the XGB model based on 5-fold cross-validation of verification set data. ROC curve, receiver operating characteristic curve; AUC, area under curve; XGB, XGBoost.

Further results suggest that the XGB model was superior with an average AUC of 0.89 (Figure 2A) and 0.79 (Figure 2B) based on training set data and verification by 5-fold cross-validation. Meanwhile, the probability of cognitive impairment predicted by the predictive model was positively correlated with the actual probability of cognitive impairment, and the model had a good degree of calibration (*P* > 0.05).

### 3.6. SHAP analysis of the model

**Figure 3:**
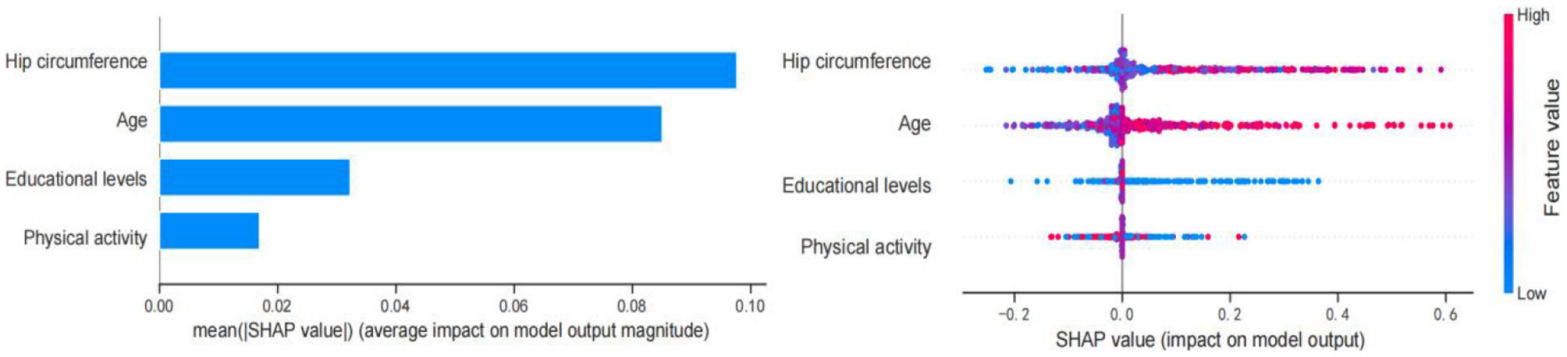
Feature importance based on SHAP results. The vertical axis shows the features, the horizontal axis represents SHAP observations. Points were colored differently with reference to their eigenvalues, pink indicating a positive correlation with early cognitive decline, and blue indicating a negative correlation with early cognitive decline.

Figure 3 shows the SHAP values for the combination of feature importance and feature effects for all individuals based on the XGB model. Each point in the diagram represents a feature and Shapley value, which represents the contribution of each feature to the predicted model output. Feature values are shown in color, and feature importance is arranged from top to bottom along the Y axis. Current SHAP results suggest that hip circumference is the most important feature in predicting Shapley value. Increased hip circumference was positively correlated with Shapley value, and larger hip circumference was more likely to be predicted as cognitive decline. Secondary to the hip circumference is age. Having an older age (colored in pink) was associated with Shapley values and was a positive predictor of early cognitive decline. Having a lower educational level and physical activity (colored in blue) was related to Shapley values and were negative predictors of cognitive decline. Overall, SHAP analysis showed that hip circumference and age were positive predictors of cognitive impairment, while educational levels and physical activity were negative predictors of cognitive impairment.

**Figure 4:**
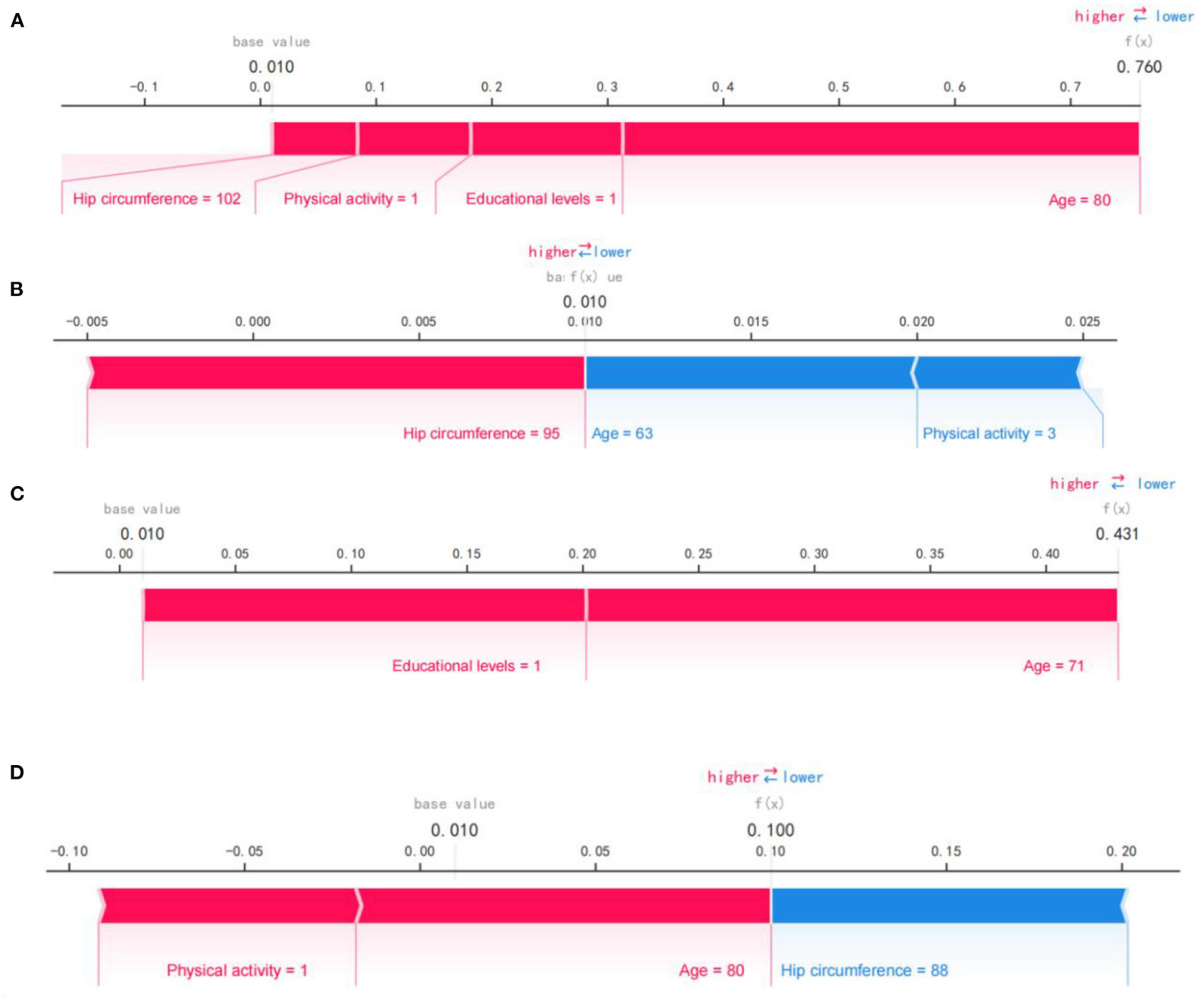
SHAP force plot for predicting early cognitive decline. **(A)** SHAP forces plot to correctly predict early cognitive decline. **(B)** SHAP forces plot to correctly predict NCI. **(C)** SHAP force plot of mispredicted early cognitive decline. **(D)** SHAP force plot of mispredicted NCI. Pink represents predictors of early cognitive decline, while blue represents predictors of NCI. Bold values show the likelihood of early cognitive decline in the ensemble model.

Figure 4 shows the SHAP force plot for predicting individual early cognitive impairment. We presented several random cases, including correct prediction and incorrect prediction. Figure 4A shows the SHAP force plot to correctly predict cognitive decline; the predictive model was supported by the Shapley value of larger hip circumference, older age, lower physical activity, and educational levels, and had a predictive probability of 0.760. Figure 4B shows the SHAP force plot to correctly predict NCI; the prediction model was supported by the Shapley value of larger hip circumference, younger age, and more vigorous physical activity, with a prediction probability of 0.990. Figure 4C shows the SHAP force plot of mispredicted early cognitive decline; the prediction model was supported by the Shapley value of higher education levels and older age, with a prediction probability of 0.431. Figure 4D shows the SHAP force plot of mispredicted NCI; the prediction model was supported by the Shapley value of more vigorous physical activity and older age, with a prediction probability of 0.900.

### 3.7. DCA modeling analysis

**Figure 5:**
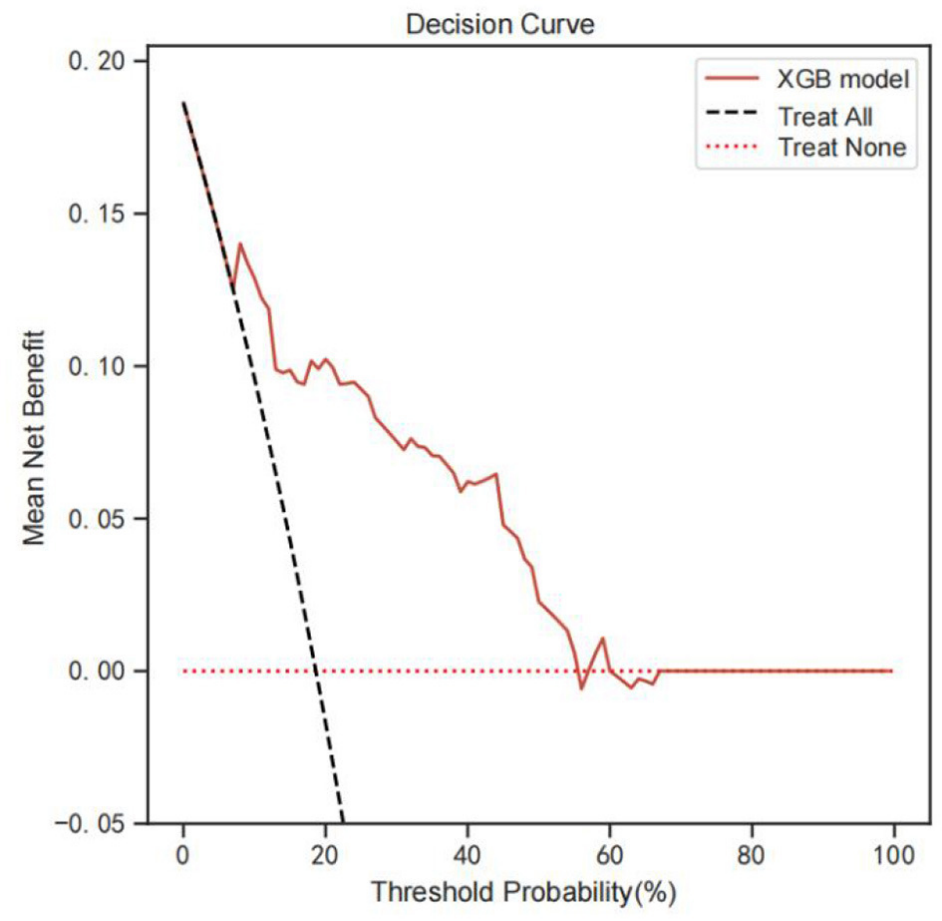
DCA analysis was performed to evaluate the clinical usefulness of the XGB model. The y-axis indicated the net benefit; the x-axis indicated the threshold probability. The solid red line shows the net benefit rate of the XGB forecast model. Within a certain threshold range, the XGB model has a higher net benefit. DCA, Decision curve analysis.

Figure 5 shows the DCA analysis results based on the XGB model. DCA analysis indicates that the XGB model had significant net benefits for threshold probabilities at different time points, suggesting the model's potential clinical benefit.

### 3.8. Visualization of the prediction model

**Figure 6:**
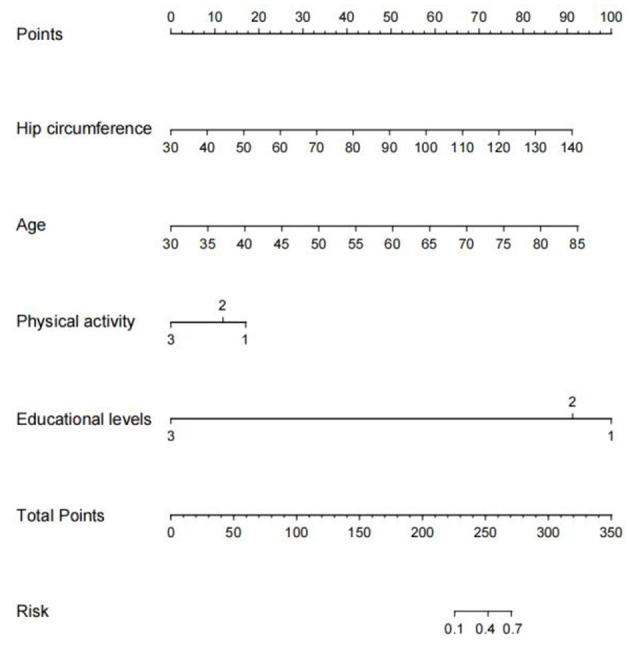
Nomogram construction for early cognitive impairment in hypertension. We established a nomogram based on the four high-risk predictors for early cognitive impairment in hypertension. In this plot, to use the nomogram model, a single node value is loaded on each variable axis and the line is drawn upwards to determine the number of points. Then, the sum of these numbers is located on the total point axis, and the line is drawn downwards to the risk of early diagnosis of cognitive impairment.

As shown in Figure 6, we developed a nomogram to predict the risk of early cognitive impairment in hypertension using four predictors, including hip circumference, age, educational level, and physical activity. The longer the line length, the greater the risk factors for early cognitive impairment. In the nomogram, each predictor has corresponding “points”, and add the points of four predictors to get the total score. Based on the total score, we can obtain the corresponding percentage risk value to determine the risk of early cognitive impairment in hypertension.

## 4. Discussion

The main findings of this study indicate that hip circumference, age, education, and physical activity were core predictors of early cognitive impairment in hypertensive individuals. Three machine learning predictive models (LR, XGB, and GNB) for early cognitive impairment based on multiple predictors in hypertensive individuals were developed and evaluated. The XGB model has the most superior predictive performance, with AUC (0.880), F1 score (0.589), accuracy (0.806), sensitivity (0.835), and specificity (0.798). Therefore, this study may provide a useful perspective for individualized accurate prediction of early cognitive impairment in the hypertensive population.

In the current study, we used LASSO regression to model feature selection. Compared to ordinary least squares regression, LASSO regression provides better control for multicollinearity and overfitting between variables and is considered holoholic to help select effective predictors of early cognitive impairment in hypertension (35). Current findings demonstrate that hip circumference, age, educational levels, and physical activity were significant predictors of early cognitive impairment in hypertension and were determined for use in machine learning model development. The SHAP analysis further determined the feature importance of four influencing factors and explained the variables involved in modeling.

Current findings suggest that hip circumference was considered the most important predictor of cognitive impairment. In this regard, we know that while the effects of obesity on the risk of cognitive impairment are well established (36), specific obesity-related indices and their relationships are still a subject of debate. Interestingly, BMI, waist circumference, and several lipid parameters were removed from the LASSO models and baseline comparison in our study. While there is evidence that these removed markers are associated with the development of cognitive impairment, several studies have reported findings similar to ours. Three previously published studies have indicated that BMI is associated with cognitive function (37–39); specifically, for every 1 kg/m2 increase in BMI, the prevalence of cognitive impairment increased by 3% (40), while another study reported the opposite result (41). A previous study based on large population data revealed a potential relationship between several obesity-related indicators, including WC, waist-to-hip ratio (WHR), BMI, LDL-C, and cognitive impairment (42). Although WC has been reported in two studies as an important indicator of cognitive ability (43, 44), current data analysis is more inclined to recommend hip circumference as a predictor of early cognitive impairment in hypertension. Although the relationship between WHR and cognitive impairment has been studied (45), the association between hip circumference and cognitive function has not been reported. Anatomically, hip circumference not only represents fat distribution, but also reflects changes in gluteal muscle, bone structure (pelvic width), and subcutaneous gluteal fat (46), which may be influenced by lifestyle-related factors such as alcohol consumption, smoking, and physical activity and other factors (47). Aging is a secondary risk factor for predicting cognitive impairment in hypertension and is considered a natural and uncontrollable factor. Moreover, it has been shown that advanced age is the main independent risk factor for cognitive impairment (48). A cross-sectional study from Shandong Province, China, showed that age was associated with cognitive impairment, but was considered a protective factor (32). This is different from our current results. Heterogeneity of the study population is the most likely reason. Our study population was a more center-based cohort of hypertensive hospitalized patients aged 30–85, and included older community adults 65 and older in the analysis. In fact, it is currently accepted that there is a consistent link between increased blood pressure and cognitive decline in middle age, but the link between blood pressure and cognitive ability is less consistent in older adults (49). Evidence that educational attainment and long-term education have a positive effect on cognitive function, especially in adulthood (50), provides a plausible explanation for the current finding that low educational attainment is a significant risk factor for early cognitive impairment in hypertension. A recent multicenter observational study based on Japanese hospital data also reported similar results to our research (51). Hypertension is often accompanied by lifestyle changes, and in the current study design, we focused on more lifestyle factors in the study population, such as sleep quality and physical activity. Surprisingly, however, the LASSO regression chose physical activity over sleep quality as a predictor of early cognitive impairment, although new evidence is emerging regarding sleep interventions in MCI and Alzheimer's disease (AD) (52). In addition, high dietary salt (53), excessive smoking, and drinking (48) may also increase the risk of cognitive impairment, but this is different from our current findings. There is accumulated evidence that physical activity may delay the progression of MCI to dementia (54), but there is also evidence that moderate to high-intensity physical activity is not beneficial in patients with early dementia (55). Herein, we observed that physical activity, as a modifiable risk factor, was analyzed as a fourth important factor in modeling early cognitive impairment in hypertension and that low-intensity physical activity was associated with the risk of developing early cognitive impairment in hypertension, supported by other earlier studies (56). Notably, SBP and DBP did not enter our model. A Mendelian randomization (MR) study noted that in middle age, high blood pressure, especially SBP, is causally associated with cognitive decline (57); it may be speculated that this result may be related to the larger number of elderly patients with hypertension included. Collectively, risk factors for cognitive impairment remain controversial. Therefore, further longitudinal analyses are needed to investigate the relationship between hip circumference, age, educational level, physical activity, and early cognitive impairment, and to further verify its ability to predict early cognitive impairment of hypertension.

Machine learning, a sub-field of artificial intelligence, is a systematic process of learning and training from data and accurately predicting the occurrence of future events (58). Recently, some scholars have developed predictive models based on machine learning for cognitive impairment, but not for hypertensive individuals. Casanova et al. (59) recommended predictors of cognitive impairment were education level, age, sex, stroke, neighborhood socioeconomic status (NSES), diabetes, APOEε4 carrier status, and BMI; distinguishing the highest and lowest grades produced the best radio frequency performance: accuracy = 78% (1.0%), sensitivity = 75% (1.0%), specificity = 81% (1.0%). Kang et al. (60) developed and validated the Aβ positive predictive model for amnestic mild cognitive impairment (aMCI) using two-stage modeling based on machine learning with good accuracy (AUC: 0.892). Tan et al. (6) used three classifiers (logistic regression, support vector machine, and gradient enhancer) to construct a set model for predicting cognitive impairment, with F1 score (0.87), AUC (0.80), accuracy (0.83), sensitivity (0.86), and specificity (0.74). In this study, we used three classifiers (LR, XGB, and GBN) to develop machine learning predictive models for early cognitive impairment in hypertension for the first time and obtained stable predictive performance. Current results suggest that the XGB model had the best predictive effect, which was better than the LR model and GBN model, with AUC (0.88), F1 score (0.59), accuracy (0.81), sensitivity (0.84), and specificity (0.80). Compared to the studies that have been reported, it seems that the elements of our model are more economical, convenient, and suitable for popularization. In addition, we further performed SHAP analysis to identify predictors that contribute most to early cognitive impairment in hypertension prediction and enhanced the interpretability and transparency of the current machine learning model. Finally, DCA analysis shows that the machine learning model has good clinical practicability and acceptability in hypertensive clinical settings.

## 5. Limitations and strengths

We have recognized the following limitations of current research. First, the main limitation of this study is the small number of samples, which is not conducive to the partitioning of the data set used to develop the model; cognitive ability may be affected by different age groups, and no age-stratified follow-up design was performed due to the small sample size. Second, participant selection procedures may be biased, which may lead to uneven distribution of data for analysis; some of the data came from self-reports collected through questionnaires, which may also lead to bias. Third, there appears to be a bidirectional association between hypertension and cognitive decline, with elevated blood pressure being both a risk factor for and a symptom of cognitive impairment; the data supporting model development is based on cross-sectional collection, which makes it difficult to derive potential causalities. Fourth, the Montreal Cognitive Assessment (MoCA), which was developed specifically for screening for MCI, appears to be more sensitive than MMSE in diagnosing early cognitive impairment (61). However, the machine learning prediction model developed based on Korean data shows that MMSE's cognitive impairment prediction algorithm also has a good prediction effect (62). Finally, we missed some possible features affecting cognitive function, such as individual genetic profiles (63), anxiety, and depression (64), which could have skewed the results. Richer dietary data are also needed, although we analyzed alcohol intake and average monthly salt intake. However, the current work also has several strengths. First, this work is the first to demonstrate the feasibility of using machine learning models to predict early cognitive impairment in individuals with hypertension. Second, we analyzed as fully as possible the economic and non-invasive development model of relevant variables. There are many candidate factors for auxiliary modeling, including sociodemographic factors, lifestyle factors, laboratory test parameters, imaging parameters, and drug information. Third, the current prediction model developed contains only four simple, non-invasive and cost-effective variables that are readily available even in poorly equipped clinical settings. Finally, the multi-center population data collection also reduces the bias to a certain extent and increases the reliability and universality of the machine model. Collectively, despite several limitations of the current study, it did provide a non-invasive and cost-effective way to predict the risk of early cognitive impairment in hypertension. Certainly, we warmly suggest future longitudinal studies that better confirm the predictive power of the model.

## 6. Conclusion

The XGB model based on hip circumference, age, educational level, and physical activity has good performance and may improve the outcome of early cognitive impairment in hypertensive clinical settings by providing early prediction and actionable feedback. In future studies, we will further develop and validate the current machine learning model based on other large-scale, multi-center population data.

## Data availability statement

The raw data supporting the conclusions of this article will be made available by the authors, without undue reservation.

## Ethics statement

The studies involving human participants were reviewed and approved by Ethics Committee of the Affiliated Hospital of Shandong University of Chinese Medicine. The patients/participants provided their written informed consent to participate in this study. Written informed consent was obtained from the individual(s) for the publication of any potentially identifiable images or data included in this article.

## Author contributions

HJ was the main coordinator of the project and was responsible for the design of the study. JY and XZ drafted the manuscript of this paper. FJ and HC were involved in the supervision of data collection and stratification. XZ, ZW, and JT contributed to data compilation and analysis. All authors contributed intellectually to this manuscript and have approved this final version.
